# Spinal Cord Injury due to Tumour or Metastasis in Aragón, Northeastern Spain (1991–2008): Incidence, Time Trends, and Neurological Function

**DOI:** 10.1155/2017/2478197

**Published:** 2017-07-25

**Authors:** Maayken Elizabeth Louise van den Berg, Juan M. Castellote, Jose Ignacio Mayordomo, Ignacio Mahillo-Fernandez, Jesus de Pedro-Cuesta

**Affiliations:** ^1^Department of Rehabilitation, Aged and Extended Care, Flinders University, Adelaide, SA, Australia; ^2^National School of Occupational Medicine, Carlos III Institute of Health, Madrid, Spain; ^3^Department of Physical Medicine and Rehabilitation, School of Medicine, Complutense University of Madrid, Madrid, Spain; ^4^Division of Medical Oncology, University of Colorado Hospital, Aurora, CO, USA; ^5^University Hospital Fundación Jiménez Díaz, Av. Reyes Católicos 2, Madrid, Spain; ^6^Centro de Investigación Biomédica en Red sobre Enfermedades Neurodegenerativas (CIBERNED), Madrid, Spain

## Abstract

**Purpose:**

Understanding the presentation of spinal cord injury (SCI) due to tumours considering population distribution and temporal trends is key to managing SCI health services. This study quantified incidence rates, function scores, and trends of SCI due to tumour or metastasis over an 18-year time period in a defined region in Spain.

**Methods:**

A retrospective cohort study included in-and outpatients with nontraumatic SCI due to tumour or metastasis admitted to a metropolitan hospital in Spain between 1991 and 2008. Main outcome measures were crude and age- and sex-adjusted incidence rates, tumour location and type, distribution by spinal level, neurological level of injury, and impairment ASIA scores.

**Results:**

Primary tumour or metastasis accounted for 32.5% of nontraumatic SCI with an incidence rate of 4.1 per million population. Increasing rates with age and over time were observed. Major pathology groups were intradural-extramedullary masses from which meningiomas and neurinomas accounted for 40%. Lesions were mostly incomplete with predominant ASIA Grade D.

**Conclusions:**

Increasing incidence rates of tumour-related SCI over time in the middle-aged and the elderly suggest a growing need for neurooncology health resources in the future.

## 1. Introduction

Nontraumatic spinal cord injury (SCI) accounts for a significant proportion of all SCI unit and rehabilitation admissions [1, 2], and allocation resources should take into account incidence and trends over time. Although cases of traumatic origin account for the largest proportion of SCI, and most SCI studies have been conducted in this group, nontraumatic SCI recently has received more attention [3–7]. The diverse underlying pathologies of nontraumatic SCI translate into differences in case ascertainment time, clinical outcomes, case management, and functional expectations [3, 6, 8–10]. The principal causes of nontraumatic SCI are primary or metastatic tumours. Tumour, intradural or extradural lesions, can cause a wide spectrum of SCI symptoms by either spinal cord compression or invasion and destruction of the spinal cord or its vasculature. Advances in diagnosis and treatment lead to earlier and higher detection of cancer and central nervous system involvement as well as to increased survival rates [8]. Subsequently, neurooncology units will increasingly be confronted with frequent admissions of patients presenting SCI due to tumour or metastasis.

Although demographic observational reports on SCI due to tumour or metastasis have been published, there is a lack of studies on incidence and trends over time. They are needed to manage allocation and delivery of health resources in population-based neurooncology settings. In order to address this goal, description of disease frequency taking into account population distribution and temporal trends is needed [9, 10].

This study aims to quantify incidence rates, functional scores, and trends of SCI due to tumour or metastatic origin in Aragón, a well-defined autonomous region in Spain over an 18-year period, and thereby seeks to anticipate for healthcare and rehabilitation resource needs in this group of patients. Specific research objectives addressed were the description of (1) incidence rates of SCI by age and sex, (2) causes of injury, and (3) severity and impairment level injury.

## 2. Methods

In Spain, patients are treated and followed up mainly in the region where they live due to the decentralization of health services. If an illness onset occurs outside the region, ultimately the patient is transferred back to the region of residence for follow-up. There are 13 specialized SCI units in the Spanish National Health System. Hospitals in the catchment area that take care of patients with SCI as secondary diagnosis, which is the case with tumoural SCI, send them to the Miguel Servet University Hospital at least for routine clinical data monitoring. That hospital is a 1200-bed public hospital in Northeastern Spain. The SCI unit of such centre is the only specialized SCI unit for up to two million inhabitants, the population of the administrative region of Aragón. Records from the SCI unit of all in- and outpatients with nontraumatic SCI due to tumour or metastasis between January 1991 and December 2008 were retrospectively reviewed. These records included referrals from other hospital units. Additionally, to capture cases not registered within the SCI unit, hospital archives and central databases from the hospital were examined using the CIE-9-MC classification.

Specification of SCI patient demographics, causes of damage, neurological level, and ASIA functional classification were extracted from the records. Additionally, age- and sex-specific population data for Aragón for each year from 1972 up to and including 2008 were obtained from the Statistics National Institute, maintained by the Government of Spain [11].

The data were divided into two time intervals, 1991–2000 and 2001–2008, to ease the observation of general trends over time. Incidence cases were grouped according to sex, age at the time of injury, and decade. Crude incidence rates were calculated within the range of 0–80 years using 10-year units and for each time interval. In addition, population denominators from the Statistics National Institute were used to construct annual age- and sex-specific incidence rates of SCI. The midyear population census for each year was used for aggregated data. Finally, in order to control for the effects of different population age distributions, crude incidence rates were age- and sex-adjusted to the European population to facilitate comparison with other studies.

The study protocol conforms to the ethical guidelines of the 1975 Declaration of Helsinki and was approved by the local health research ethics board of the Carlos III Health Institute, Madrid, Spain (2008/01).

## 3. Results

A total of 277 nontraumatic cases were identified during the 18-year period studied, from which 90 (32.5%) were due to primary tumour or metastasis as shown in Table 1. The most common primary cancer sites for metastasis were lymphoma, as well as breast, lung, and prostate. Just over half of the cases (56.6%) were women. Mean age at diagnosis for women was 52 years for the first period (1991–2000) and 62 years for the second period (2001–2008), that is, approximately raising a year per calendar year. For men it was 53 years for the first period (1991–2000) and 58 years for the second period (2001–2008), an increase 50% lower than for women.

**Figure 1:**
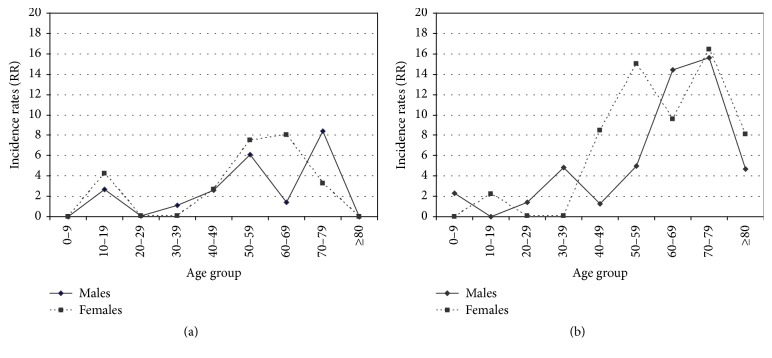
Age- and sex-specific incidence rates per million population in study periods 1991–2000 and 2001–2008. (a) Period 1991–2000. (b) Period 2001–2008.

The incidence rate adjusted by sex and age, covering the whole study period, was 4.1 per million population: 3.6 per million for men and 4.6 per million for women. Incidence rates in the second period (2001–2008) almost doubled with respect to the first period (1991–2000); see Table 1. Although generally incidence increased with age, patterns were different for men and women. In the second period (2001–2008), for men, the strong increase in incidence was observed over 50 years of age, but for women this increase occurred ten years earlier at the age of 40 (Figure 1). The increase in cases from the first (1991–2000) to the second period (2001–2008) was higher among women.

**Figure 2:**
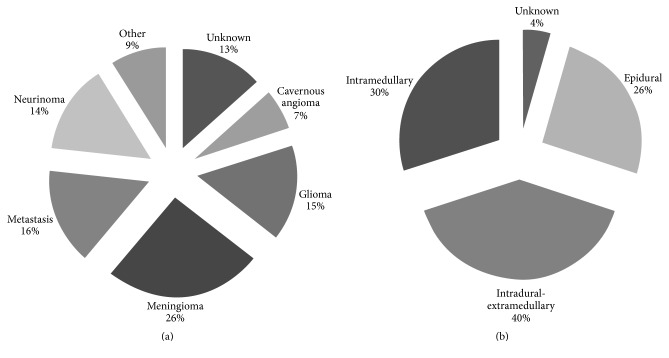
Anatomical location and histopathology classification of spinal cord tumours and metastases. (a) Anatomical location. (b) Histopathology classification.

According to their location, most tumours and metastasis were located intradural-extramedullary (Figure 2(a)), including meningiomas (26%) and neurinomas (14%) (Figure 2(b)). Extradural metastasis (mostly from carcinomas of the breast and prostate and from lymphoma) and intramedullary gliomas accounted each for 15.6% of the cases. Angiomas were distributed intramedullary and accounted for 6.7% of all cases. The increase between periods for women was remarkable for meningiomas (360% increase) and neurinomas (260% increase). There has been a reduction of gliomas and angiomas over time although overall number of cases has been small (14 and 6, resp.).

**Figure 3:**
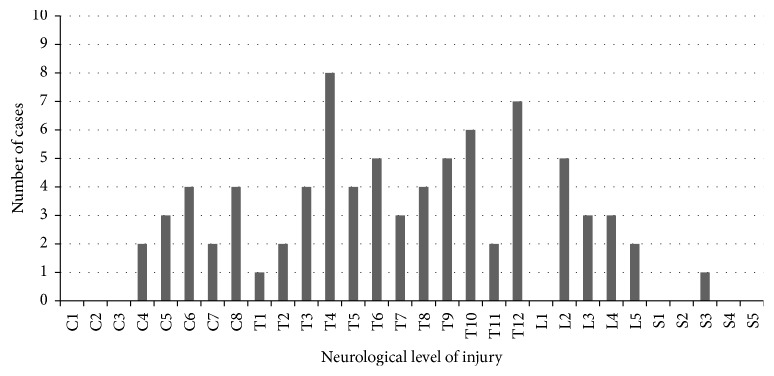
Number of SCI cases by neurological level of injury.

**Figure 4:**
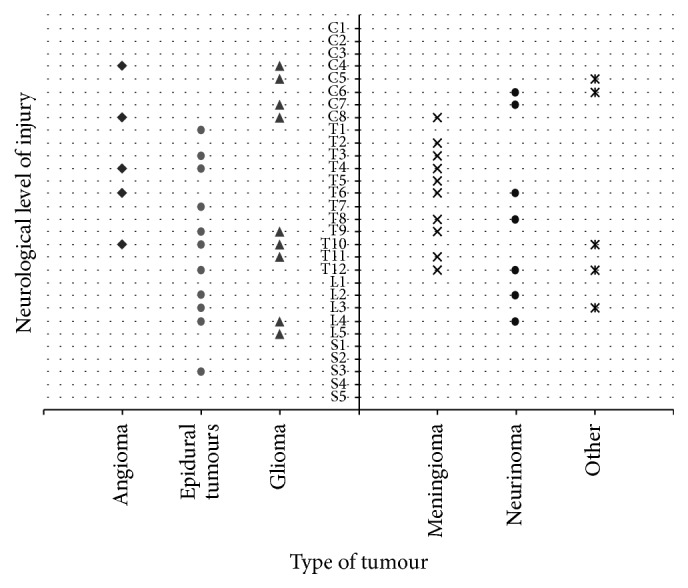
Type of tumour, metastases, and neurological levels affected. Epidural tumours are not split due to the small size of subcategories.

Location of damage was variable across spinal cord levels. Frequency distribution in Figure 3 shows that injury mainly occurred at the thoracic segments (56.7%), with frequency peaks at T4 and T12. Cervical lesions accounted for 17.8% of the cases and lumbar and sacral lesions for 14.4% and 1.1% of the cases, respectively. In 10% of the cases the location was unknown. As seen in Figure 4, lesion location varied according to the histopathology of the tumour or metastasis. Epidural tumours, mainly metastasis, were situated mostly at thoracic and lumbar levels and meningiomas at thoracic level. Intramedullary gliomas were found at cervical, low-thoracic, and lumbar levels. Other tumours were distributed over the full length of the spinal cord.

**Figure 5:**
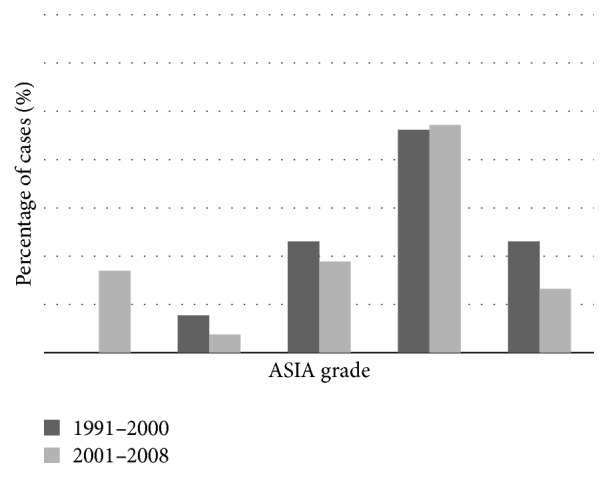
ASIA classifications in individuals with nontraumatic SCI due to tumour or metastasis. ASIA scores: Grade A: complete: no motor or sensory function is preserved in the sacral segments S4-S5. Grade B: incomplete: sensory but not motor function is preserved below the neurological level and includes the sacral segments S4-S5. Grade C: incomplete: motor function is preserved below the neurological level, and more than half of key muscles below the neurological level have a muscle grade less than 3. Grade D: incomplete: motor function is preserved below the neurological level, and at least half of key muscles below the neurological level have a muscle grade of 3 or more. Grade E: normal: motor and sensory function are normal.

Functional ASIA score was recorded in 73% of the cases with most missing data in the first period (1991–2000). Distribution of scores for incomplete lesions followed a shifted bell-shaped curve with highest numbers in Grade D (47%) as seen in Figure 5. Complete lesions (ASIA Grade A) were found in 13.6% of the patients.

## 4. Discussion

In the present study, we describe incidence, neurological function, and time trends of SCI due to tumour or metastasis in Aragón, Spain. From the first (1991–2000) to the second (2001–2008) studied time period, incidence rates more than doubled. Additionally, incidence rates increased with age in both males and females. The results suggest that the increase in females is mainly due to higher numbers of meningiomas and neurinomas. Neurological function was not greatly affected, possibly since most cases presented with incomplete lesions in both time periods.

The comparability of incidence rates of tumoural SCI in Spain with other countries in and outside Europe is problematic due to lack of such information in the literature. Reports commonly are retrospective case-series from medical centres or case reports describing number of cases and studied period, but typically not catchment area or with lack of demographical data. Therefore, medical articles mainly reporting clinical data omit a potential epidemiological perspective [12–15], hence making a limited contribution to the implementation of health services for individuals with SCI due to tumour or metastasis. This population-based study, covering an 18-year time period, is novel showing age- and sex-adjusted incidence rates and trends of tumoural SCI.

Proportions of primary and secondary tumours differ from previous reported results: McKinley et al. [1] showed that 85% of the lesions were due to metastatic tumours and in an Italian recent report [15] this was 24%, compared to 16% in the present study. Possibly these differences can be explained by the above-mentioned discrepancy in data reporting. One additional explanation may well be the increased awareness among physicians of the devastating effects of SCI in cancer patients. This has resulted in more frequent use of palliative radiation therapy and bisphosphonates in those with spinal metastases leading to reduced SCI involvement.

Previous reports [14, 15] have observed that in individuals with tumoural SCI a higher proportion of lesions affect the thoracic levels, which is consistent with our study results. However, one should bear in mind that anatomically there are more thoracic metameric levels than lumbosacral or cervical ones. The data revealed that the site of lesion varied according to the cause. In the present study, metastasis and meningiomas accounted for most cases. In Spain it is known that the most frequent tumours are those due to lung cancer in men and breast cancer in women and that incidence rates of the later have increased over the last decades [16]. However, we have seen that rates of spinal cord metastases including those of breast cancer have not changed much over the studied time periods. Rates that did raise over time, mainly observed in women, were due to meningiomas, involving primarily thoracic levels, and neurinomas. Although the study period covered 18 years, the amount of data is too little to draw firm conclusions about a univocal relationship between tumour type, metastasis in spinal cord, and changes over time.

Previous research findings have reported that between 20% and 32% of the nontraumatic SCI cases correspond to SCI due to tumours or metastases [6, 15, 17–20]. Our study covered a well delimited health area in Spain and covering a period of 18 years we identified 90 tumoural SCI cases, corresponding to 33% of nontraumatic cases. The fact that our study results show slightly higher proportion of tumoural SCI than previous research studies [6, 15, 17–20] may be due to the extensive case ascertainment. We should stress that this study included all consecutive cases that were followed by the specialized SCI unit including those registered through referrals from other hospital units. Additionally, patients staying in wards other than the SCI unit were tracked by searching hospital archives. It is known that few cancer patients with SCI are admitted to spinal cord units [12]. We cannot discard possible missing cases in which spinal cord damage or symptoms are not so evident; for example, the existence of comorbidities may hamper diagnosis. Epidemiological reviews have shown that nontraumatic SCI affects mainly people aged 50 years and older and that incidence rates increase with age in both males and females [21–23]. In the present SCI study concerning tumours we have observed a similar trend. In contrast, a different incidence rate pattern has been demonstrated in SCI in the same population due to traumatic causes such as traffic accidents or falls, with a peak in young adults and in general higher rates for men [22]. Acute deterioration with short survival in individuals with SCI due to tumours has been well described [14, 22, 24, 25]. On the other hand, in SCI due to traffic accidents or falls, among the most frequent causes, the survival rate nowadays is close to that of healthy subjects [22]. This supports the study of traumatic and tumoural independently from traumatic SCI cases, in order to better plan, develop, and assess use of health resources by this group of patients.

ASIA Grades A and B were the least frequently reported scores in both time periods, suggesting that function in tumoural SCI typically is partially preserved. This corresponds with findings from previous reports [13, 15]. It seems that, in general, function following tumoural SCI is better conserved than in SCI due to other causes, either traumatic or nontraumatic [7, 15]. In our population-based study we observed a high proportion of ASIA Grade D. Eriks et al. [12] found ASIA Grade D to be prognostic for prolonged survival (>1 year after discharge). ASIA Grade D rates in the present study, higher than in some other studies [13–15], may be explained by the inclusion of all consecutive identified SCI cases in the present study compared to selected samples in other studies.

Unfortunately, the retrospective design of the study is prone to biases including selection bias and recall bias. Taking this into account we tried to be as inclusive as possible. The study included all consecutive cases that were followed by the specialized SCI unit and those referred through other hospital units or staying in other wards were tracked down additionally; however, missing cases cannot be discarded. Up to date the paradox of the unknown incidence of metastatic epidural spinal cord compression (MESCC) remains as there is a discrepancy between the 3–5% of patients with advanced cancer with metastases to the spinal column and the postmortem incidence of 5–10% on this kind of patients [26, 27]. Additionally there is a relatively low incidence (2.9% of all primary brain and CNS tumours) of primary central nervous system tumours located in the spine and spinal cord on the other hand [28]. Difficulties in estimation of true incidence are hospital-dependent detection rates, correct diagnosis, and entry into recognised coding systems [29]. The true incidence of MESCC is unknown and can only be estimated. Many cancer patients have asymptomatic or unrecognised MESCC while others develop MESCC after the decision has been made to not undergo extensive diagnostic testing or therapy. Although the goal is to establish the diagnosis prior to the development of spinal cord damage, the diagnosis is often delayed, for example, in patients who have back pain [30] or present with weakness [31]. However, advances in diagnosis lead to improved detection and identification of SCI cases caused by tumours and metastasis. This, together with a recently developed international SCI data set for nontraumatic SCI [5], hopefully will contribute to the increasing quality of future studies assessing incidence rates and epidemiological trends.

## 5. Conclusions

Patterns of age-specific incidence rates, such those found in the present study, reveal the age groups to which special disability healthcare efforts should be directed. Our study shows that risks increase with age and that tumoural SCI is mainly observed in the middle- and old-aged. This, as well as the high mortality rates of individuals with SCI that still exceed those of the age-matched nondisabled population, pleads for a focus on short term disability healthcare goals. Additionally, it emphasizes the need for a multidisciplinary approach and a quick evaluation process regarding patient transfer to nursing home, hospice, or home. Ultimately, this work allows for the conduct of future studies focused on health outcomes such as survival and quality of life for each subtype of tumoural SCI.

## Figures and Tables

**Table 1:** Incidence and demographics of spinal cord injury due to primary tumour or metastasis in Aragón, Spain, 1991–2008. European population taken as standard for direct adjustment.

	1991–2000	2001–2008	Total
*SCI incidence*			
Number of cases	32	58	90
Crude incidence rate	2.7	5.8	4.1
Adjusted incidence rate	2.5	4.8	
Males	2.2	4.2	
Females	2.8	5.3	
*Sex*, % male	43.8	43.1	43.3
*Age*, mean (SD)			
Total	52.1 (19.5)	60.1 (17.0)	57.3 (18.3)
Males	52.5 (19.6)	57.7 (19.6)	55.8 (19.5)
Females	51.8 (20.0)	62.0 (14.9)	58.4 (17.4)

## Abbreviations

SCI: Spinal cord injury.
